# Electrochemical Impedance Spectroscopy of Anion-Exchange Membrane AMX-Sb Fouled by Red Wine Components

**DOI:** 10.3390/membranes11010002

**Published:** 2020-12-22

**Authors:** Anton Kozmai, Veronika Sarapulova, Mikhail Sharafan, Karina Melkonian, Tatiana Rusinova, Yana Kozmai, Natalia Pismenskaya, Lasaad Dammak, Victor Nikonenko

**Affiliations:** 1 Membrane Institute, Kuban State University, 149 Stavropolskaya Street, 350040 Krasnodar, Russia; vsarapulova@gmail.com (V.S.); shafron80@mail.ru (M.S.); n_pismen@mail.ru (N.P.); v_nikonenko@mail.ru (V.N.); 2 Central Research Laboratory, Kuban State Medical University, 4 Sedina Street, 350063 Krasnodar, Russia; agaron@list.ru (K.M.); rusinova.tv@mail.ru (T.R.); yana.yutskevich@gmail.com (Y.K.); 3 Institut de Chimie et des Matériaux Paris-Est (ICMPE), UMR 7182 CNRS, Université Paris-Est, 2 Rue Henri Dunant, 94320 Thiais, France; dammak@u-pec.fr

**Keywords:** electrochemical impedance spectroscopy, anion-exchange membrane, wine, anthocyanins, fouling, biofouling

## Abstract

The broad possibilities of electrochemical impedance spectroscopy for assessing the capacitance of interphase boundaries; the resistance and thickness of the foulant layer were shown by the example of AMX-Sb membrane contacted with red wine from one side and 0.02 M sodium chloride solution from the other side. This enabled us to determine to what extent foulants affect the electrical resistance of ion-exchange membranes, the ohmic resistance and the thickness of diffusion layers, the intensity of water splitting, and the electroconvection in under- and over-limiting current modes. It was established that short-term (10 h) contact of the AMX-Sb membrane with wine reduces the water-splitting due to the screening of fixed groups on the membrane surface by wine components. On the contrary, biofouling, which develops upon a longer membrane operation, enhances water splitting, due to the formation of a bipolar structure on the AMX-Sb surface. This bipolar structure is composed of a positively charged surface of anion-exchange membrane and negatively charged outer membranes of microorganisms. Using optical microscopy and microbiological analysis, it was found that more intense biofouling is observed on the AMX-Sb surface, that has not been in contacted with wine.

## 1. Introduction

Electrodialysis (ED) with ion-exchange membranes (IEM) is increasingly used for tartrate stabilization of wine and the isolation of valuable components (for example, anthocyanins, which are antioxidants and natural dyes) [1] from pulp and other waste of wine production [2,3,4,5]. The advantages of ED in comparison with baromembrane methods are in reducing the loss of valuable wine components [6,7] and the ability to effectively desalinate wine materials and juices with simultaneous reagent-free pH correction [8,9].

Note that wine materials contain more than 600 components, including polysaccharides, amino acids, proteins and polyphenols (anthocyanins, proanthocyanins, tannins, etc.) [10]. The interactions of these substances with the materials comprising the IEM and with each other cause fouling [11,12,13], which reduces the life cycle of membranes and affects energy consumption and other parameters of the ED process [14]. An analysis of works devoted to this problem allows us to conclude that the main mechanisms of fouling are: van der Waals interactions of polyphenols with materials comprising the IEM; π – π (stacking) interactions [15,16] between aromatic rings of polyphenols and the ion-exchange matrix of IEM (which, in most cases, consists of a copolymer of divinylbenzene and polystyrene). Electrostatic interactions [17,18,19] take place if the components of wine materials gain an electric charge opposite to the charge of membrane fixed groups. Indeed, in an acidic environment, anthocyanins become cations, and in an alkaline environment they are anions [20]. The colloidal state of organic molecules (formed due to the interactions between polyphenols, polysaccharides, amino acids and proteins) on the surface and in the volume of ion-exchange membranes is supported by weak van der Waals attraction forces, electrostatic repulsion forces and hydrogen bonds [11].

Nitrogen-containing fixed groups of anion-exchange membranes are a nutrient medium for microorganisms [21]. Besides this, wine materials and waste from winemaking are rich in saccharides, amino acids and other substances, the presence of which stimulates biofouling [22]. It should be noted that some microorganisms can be adsorbed at the membrane surface as early as during the first hours of membrane module operation [23]. Piping, storage tanks, pretreatment systems [24] can be a source of microbial contamination. Microorganisms such as those responsible for brewing [25] may find their habitat in these parts of apparatuses.

Electrochemical impedance spectroscopy (EIS) is increasingly being used to study IEM fouling [26,27,28,29,30,31]. The method of equivalent electrical circuits (EEC) is most often applied for the interpretation of experimental spectra [28,29,30,31]. For example, an increase in the ohmic resistance of a membrane, which is determined by processing a high-frequency EIS arc using the EEC method, is the basis for online registration of the effect of fouling on the transport characteristics of membranes in reverse and conventional ED [30,31]. If the electrical resistance (and capacitance) of foulants or modifying films used for fouling prevention and control differs markedly from similar characteristics of the pristine membrane, researchers observe the appearance of additional arc in the high-frequency domain of the impedance spectra [28,29,30,31]. In this case, processing the high-frequency domain of impedance spectra using EEC gives information on the resistance, capacitance and thickness of the fouling or modifying layers. It is shown in [32] that an increase in the membrane surface fouling degree by sodium dodecyl sulfate is accompanied by an increase in the high-frequency arc of impedance spectra, as well as a decrease (and, finally, the disappearance) of the low-frequency (diffusion) arc. The authors of [32] believe that the degradation of the low-frequency arc indicates an almost complete cessation of ion migration through the IEM. Park et al. [27] used the EEC method to analyze the manifestation of fouling in the mid-frequency and low-frequency EIS domains, where spectra were obtained under conditions of an applied direct current (as in electrodialysis). They found that the presence of bovine serum albumin (BSA) on the anion-exchange membrane (AEM) surface causes the appearance of an arc in the mid-frequency domain of the impedance spectra and an additional capacitive loop in the low-frequency domain (<100 Hz), which were absent in the case of the pristine membrane. Park et al. [27] suggested that the observed changes in the impedance spectra are caused by the formation of the bipolar AEM/BSA boundary, which contributes to enhanced water splitting. To approximate these spectra, they introduced into the equivalent circuit an additional parallel element of resistance and capacitance to take into account the appearance of a BSA layer on the AEM surface. Besides, they replaced the capacitor, indicator of Warburg impedance, on the inductor, which had to take into account the chemical reaction (water splitting) in a parallel element that described transport phenomena in diffusion layers.

In a number of works [33,34,35], it was theoretically and experimentally shown that the mid-frequency arc (Gerischer-type impedance) is primarily an indicator of water-splitting at the IEM/solution boundary or at the bipolar interface. The low-frequency arc (Warburg-type impedance), first of all, characterizes the ion diffusion transport in the solution layers adjacent to the IEM and can be used to evaluate the influence of various factors (for example, the development of electroconvection) on the diffusion layer thickness [36,37].

In this work, we applied both the mathematical models of EIS and EEC method to interpret the impedance spectra in the full frequency range. By the example of AMX-Sb anion-exchange membrane contacted with red wine, we will demonstrate the capabilities of the EIS for the first time, for a comprehensive study of the fouling and biofouling effect, not only on the characteristics of AEMs, but also on their behavior under conditions of ED desalination of a NaCl solution.

## 2. Experiment

### 2.1. Membranes and Solutions

The homogeneous anion-exchange membrane AMX-Sb (Astom, Tokyo, Japan) was selected as the object of study. This membrane is manufactured by paste method [38]. It contains an inert filler: granules of polyvinyl chloride, whose diameter reaches 60 nm. The matrix of the ion-exchange material of the AMX-Sb consists of a copolymer of styrene and divinylbenzene. The fixed groups are mainly quaternary ammonium bases [38].

The main characteristics of the membrane under study are presented in Table 1.

In the experiments we used: distilled water (electrical conductivity was 0.5 μS cm^−1^, pH = 5.5, 25 °C), solid NaCl of analitycal grade (JSC Vekton, St. Petersburg, Russia), red dry wine made from the varieties of the Murvedr, Syrah, Grenache grapes (pH = 3.5).

### 2.2. Methods

#### 2.2.1. Membrane Fouling Procedure

**Figure 1:**
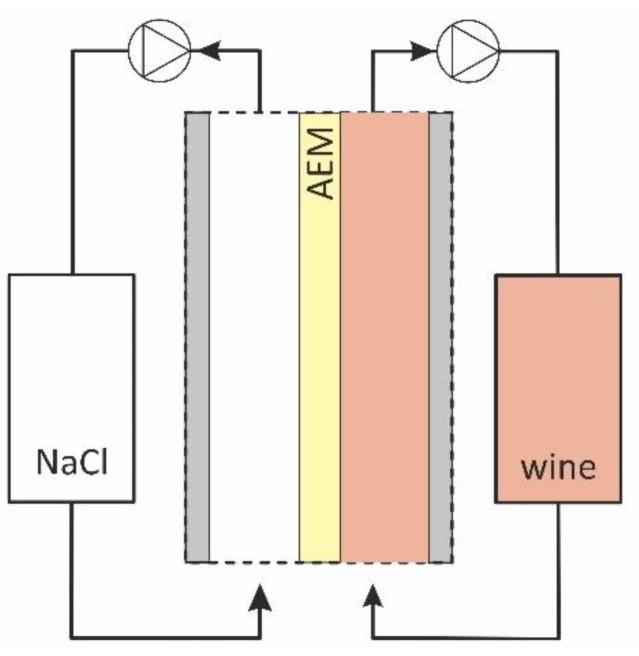
Scheme of the flow-cell used for AEM fouling.

Before the study, all membrane samples underwent standard salt preparation [40] and were equilibrated with 0.02 M NaCl solution. One of these samples was used for comparison. Other samples (indicated by the subscript “w”) were placed in a two-compartment flow cell (Figure 1).

The 0.02 M NaCl solution was circulated through one of the compartments and the red wine through the other. The time of the sample contact with wine in hours is indicated by a subscript. Thus, “AMX-Sb_w10_” denotes the sample which was in contact with wine during 10 h; “AMX-Sb_w72_” relates to the sample which was in contact with wine during 72 h. In the course of the experiment, no special measures were taken to prevent the ingress of microorganisms into the wine and NaCl solution circuits in order to simulate conditions favorable for biofouling of the studied membrane. As already mentioned in the Introduction [11], such conditions often arise during the ED processing of food industry liquid media.

After the completion of the fouling stage, the concentrations of ethyl alcohol and glucose were determined by liquid chromatography [41] and spectrophotometry [42] methods.

#### 2.2.2. Visualization

The visualization of surface and cross-sections of swollen AMX-Sb membranes before and after fouling was carried out using a SOPTOP CX40M optical microscope (Yuyao, China) with a set of 5×, 10×, 20×, 50× and 100× objectives and a digital eyepiece camera.

#### 2.2.3. Microbiological Analysis

After 72 h of contact with wine, the studied sample was removed from the cell (Figure 1) and placed in a sterile Petri dish. Touch smears of the AMX-Sb surface exposed to NaCl solution and of the surface exposed to wine were made on defatted glass slides. The obtained preparations were dried in air, fixed over the flame of an alcohol lamp, and then stained by the Gram-staining method [43] in the following sequence: 2–3 drops (50–75 μL) of carbolic solution of gentian violet for 2 min; 2–3 drops of Lugol’s solution for 1 min; discoloration with 96% ethyl alcohol for 30–45 s; rinsing with distilled water; 2–3 drops of an aqueous solution of fuchsin for 2 minutes; rinsing with distilled water.

Gram-positive microorganisms turned blue-violet, and gram-negative ones turned pink-red. Optical images of the stained preparations were performed using a Primo Star microscope, Carl Zeiss, Oberkochen, Germany, with a set of 10×, 100× in the presence of immersion liquid. Similar manipulations were made on the pristine AMX-Sb membrane just after salt pretreatment procedure.

#### 2.2.4. Electrochemical Impedance Spectroscopy

**Figure 2:**
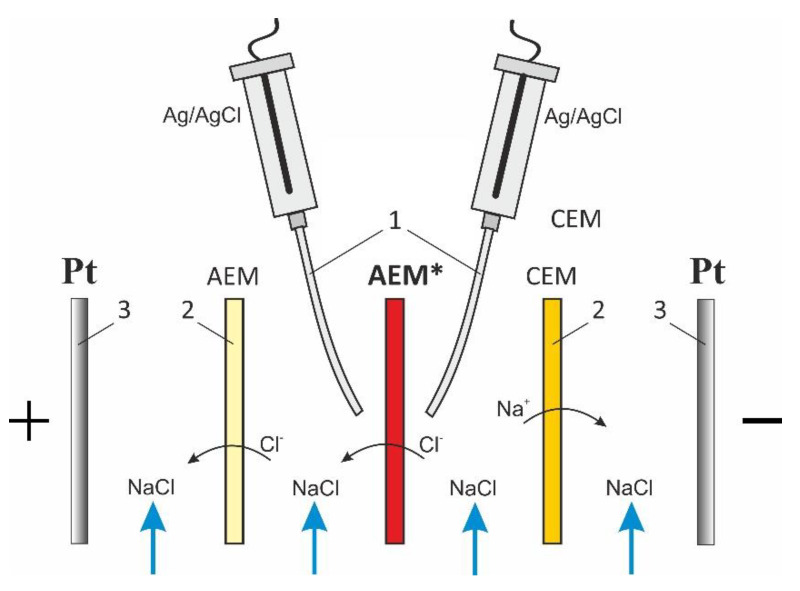
Scheme of the membrane system under study. In figure: AEM* is the anion-exchange membrane under study, the Luggin capillaries (1), two auxiliary membranes, an anion-exchange and a cation-exchange ones (2), platinum polarizing the working and counter electrodes (3).

The spectra of the electrochemical impedance were measured in a flow electrochemical cell using the Autolab PGStat-100 electrochemical complex. The desalination compartment was formed by the investigated anion-exchange membrane AMX-Sb (Astom, Japan) and the auxiliary cation-exchange membrane MK-40 (UCC Schekinoazot, Pervomayskiy, Russia). Installation, as well as the method of data-obtaining and processing, are described in detail in [44]. The scheme of the studied membrane system is presented in Figure 2.

The intermembrane distance, *h*, is 6.5 mm; the linear flow velocity of the 0.02 M NaCl solution, *V*, is 0.4 cm s^−1^; the area of the polarized section is 2 × 2 cm^2^. The AMX-Sb_w_ was oriented towards the desalination compartment with a surface that was in contact with the wine. Before the measurements, AMX-Sb_w_ samples were preliminarily soaked in a 0.02 M NaCl solution for 24 h. The investigations were carried out at a temperature of 20 ± 1 °C and various values of $i/i_{\lim}^{theor}$.

The limiting current density, $i_{\lim}^{theor}$, was calculated from the Lévêque equation obtained in the framework of the convection-diffusion model [45]

(1) $$i_{\lim}^{theor}=1.47\frac{FDC}{h(T_{Cl}-t_{Cl})}{\left(\frac{h^{2}V}{LD}\right)}^{1/3},$$

where *L* is the length of the desalination compartment; *C* is the molar concentration of NaCl at the entrance to the desalination compartment, $T_{Cl}$ and $t_{Cl}$ are the transport numbers of chlorine ion in the membrane and in the solution, respectively; *D* is the diffusion coefficient of NaCl at infinite dilution; *F* is the Faraday constant.

The limiting current density, calculated using Equation (1) for the membrane system under study, is equal to 2.9 mA cm^−2^.

The time for obtaining each electrochemical impedance spectrum is about two hours: at first, the membrane is held for 20 minutes at a given direct current, then equilibrated at each given frequency (from 3 × 10^−3^ to 1.3 × 10^5^ Hz), starting from the low frequencies.

Spectra are obtained passing from lower current densities, *i*, to higher ones. The time intervals between the measurements in the absence of direct current (*i* = 0) are 40 min. The impedance of a membrane and adjoining diffusion boundary layers (DBLs) was determined by subtracting spectra measured with a membrane and without it at corresponding frequencies [36].

**Figure 3:**
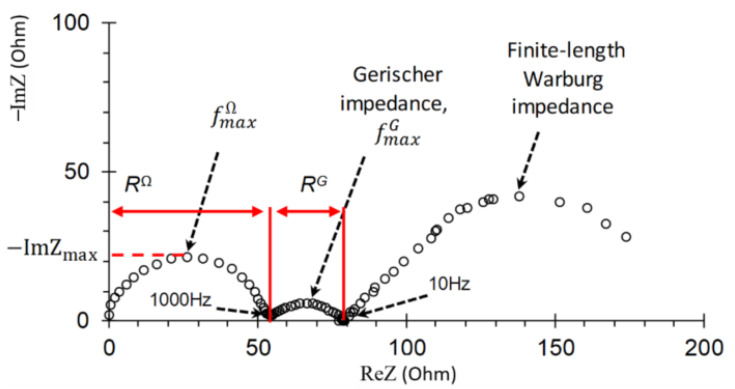
Typical impedance spectrum of a monopolar ion-exchange membrane and adjacent diffusion layers in the over-limiting current mode.

A characteristic impedance spectrum of an IEM in a solution of strong electrolyte is shown in Figure 3. It is represented on the complex Argand plane and includes three arcs. The first arc appears at high frequencies (in the range from 10^3^ to 1.3 × 10^5^ Hz). Its shape is mainly determined [46,47] by electrical capacitances and ohmic resistances of the layers in the membrane system under study (DBL/membrane/DBL). The width of the high-frequency arc is equal to *R*^Ω^, which is the ohmic resistance of the membrane and the adjacent DBLs. The maximal value of the imaginary component of impedance on this arc, −ImZmax, and the frequency corresponding to this value, $f_{\max}^{\Omega }$, is used to estimate the effective capacitance according to equation [46,48]

(2) C=14πfmaxΩ(−ImZmax)

This capacitance includes the capacitances of the electric double layers (EDL) on the membrane/solution boundaries, as well as the capacity caused by the asymmetry of the depleted and enriched DBLs, which occurs when a direct electric current flows. The capacity of the EDL is dominant in the given (10^3^–10^5^ Hz) frequency range [47].

The mid-frequency (10–10^3^ Hz) arc of so-called Gerischer impedance is observed when additional charge carriers appear in a system as a result of the chemical reaction (in case of the considered membrane system, the water-splitting reaction at the membrane/solution boundary) [35,46]. The width of this arc is equal to the effective resistance of the reaction layer, *R^G^*.

The frequency corresponding to the maximal value of imaginary component of the Gerischer impedance spectrum, $f_{\max}^{G}$, is used to calculate the effective water-splitting reaction constant on the membrane/solution boundary [35]

(3) $$\chi =\frac{2\pi f_{\max}^{G}}{\sqrt{3}}$$

The low-frequency (0.003–10 Hz) arc of the spectrum (finite-length Warburg impedance) characterizes the diffusion and electroconvective transport of ions in DBL adjacent to a membrane. The DBL thickness can be found from the difference in values of the real component of the low-frequency arc for the lowest and the highest frequencies [37]. 

## 3. Results and Discussion

### 3.1. Optical Images

**Figure 4:**
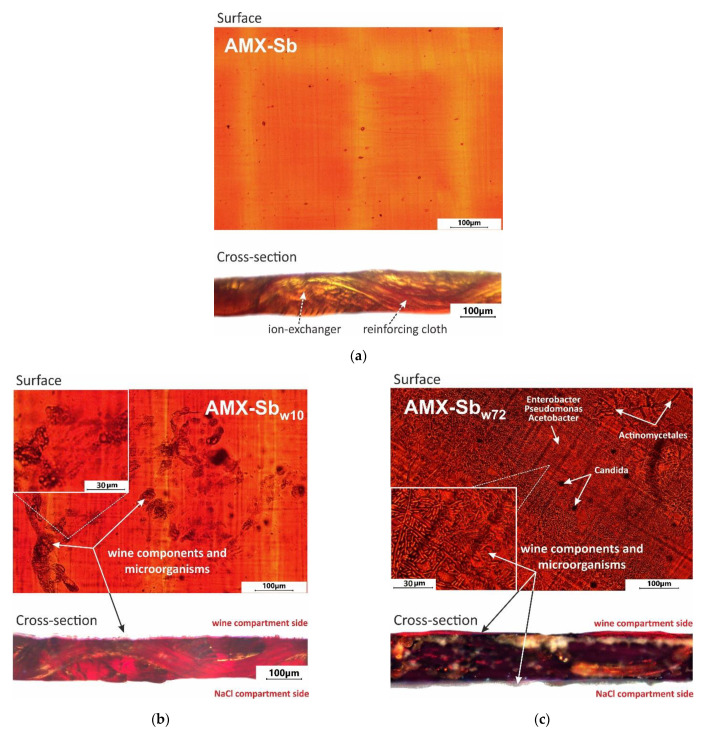
Optical images of the swollen AMX-Sb (**a**), AMX-Sb_w10_ (**b**) and AMX-Sb_w72_ (**c**) samples surface and cross-section.

The results of swollen pristine and fouled AMX-Sb membrane surface and cross-section optical microscopy are shown in Figure 4.

As can be seen from Figure 4b, after 10 h of contact with wine, the surface and cross-section of the AMX-Sb_w10_ sample acquire a pale ruby color. This color is an indicator of the penetration of anthocyanins into the membrane volume [12,13]. On the surface of AMX-Sb_w10_, structures are visualized, the distribution of which has an “island” character. These structures are mainly composed of high-molecular-weight components of wine [13].

After 72 h of contact with wine (Figure 4c), the color of the surface and cross-section of the membrane turns red-brown, indicating that the volume of AMX-Sb_w72_ sample is enriched with tannins and/or anthocyanin-tannin adducts (for example, catechins), which are yellow and brown, respectively [49]. Colored aggregates of wine components and microorganisms almost completely cover the surface of AMX-Sb_w72_, which was exposed to the wine compartment (Figure 4c). On the opposite surface of the membrane, which was facing the NaCl compartment, a translucent film is visualized. As will be shown in Section 3.2, this film is formed by microorganisms.

### 3.2. Results of Microbiological Analysis

**Figure 5:**
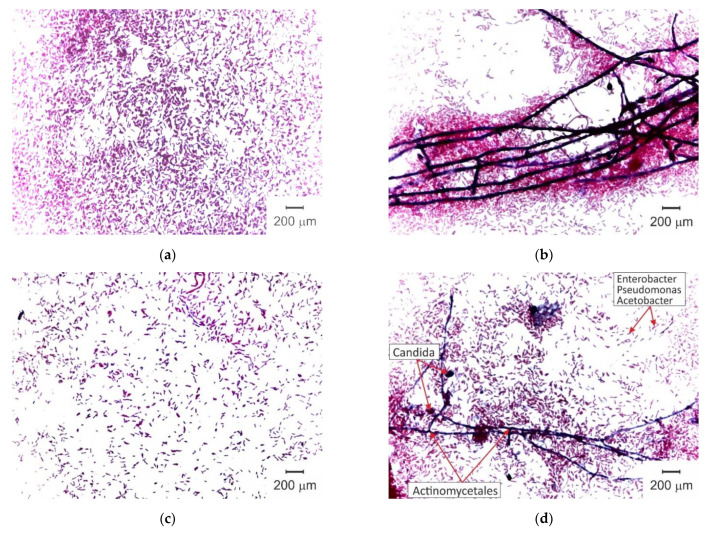
Optical images of the inoculation from touch smears taken from AMX-Sb surfaces facing the NaCl compartment (**a**,**b**) and the wine compartment (**c**,**d**).

The pristine AMX-Sb sample surfaces immediately after salt pretreatment do not contain microorganisms. Figure 5 shows the results of microbiological analysis of the surface microflora of AMX-Sb_w72_. On the surface facing the NaCl solution compartment (Figure 5a,b), gram-negative, non-spore-forming aerobic rod-shaped bacteria were found, morphologically similar to representatives of the genera Enterobacter, Pseudomonas and Acetobacter. In addition, gram-positive bacteria Actinomycetales, which have the ability to form branching pseudomycelium, and single cells of microscopic fungi of the genus Candida, which are stained in dark purple, are visualized. The same representatives of microflora, but in much smaller quantities, were found on the surface facing the wine compartment (Figure 5c,d).

The source of microflora is most likely the elements of the ED system (hoses, etc.), as is often the case in real production. Less rapid development of microflora from the side of wine compartment is caused by the presence of ethyl alcohol, which suppresses its reproduction [50,51]. The more intensive growth of microflora on the AMX-Sb surface facing the NaCl solution compartment is apparently due to the absence of ethyl alcohol in this solution, as well as (as our measurements show) a high concentration of glucose and other nutrients at the AMX-Sb/NaCl interface, that are transported across the membrane from the wine compartment. In the future, we plan to obtain more detailed information about this phenomenon, as well as about the structure of foulant on the membrane surface, using optical coherence tomography, OCT, and fluorescence microscopy method.

The obtained results are important for the practice of ED tartrate stabilization of wine and ED separation of anthocyanins from winemaking waste. First, they confirm the legitimacy of the established electrodialysis practice, where membrane stacks in the food industry are sterilized every 10–12 h [11,52,53]. Indeed, during this time, biofouling does not have time to significantly affect the membrane transport characteristics. Secondly, these results show that it makes sense to pay more attention to the periodic sterilization of the concentration compartment (circuit), and not just the circuits of electrodialyzer with wine or other food media, as is most often done now.

### 3.3. Electrochemical Impedance Spectra

Using electrochemical impedance spectroscopy (EIS), let us analyze to what extent the changes in the volume and surface of membranes after contact with wine affect their behavior in the superimposed electric field (DC bias).

**Figure 6:**
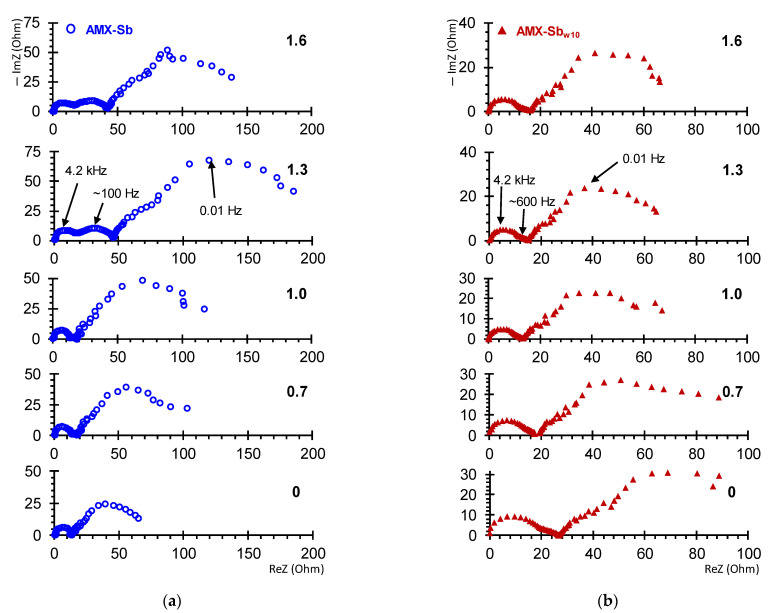
Electrochemical impedance spectra of AMX-Sb (**a**) and AMX-Sb_w10_ (**b**) samples. The numbers near the curves denote the values of $i/i_{\lim}^{theor}$.

Figure 6 shows the EIS of the AMX-Sb (Figure 6a) and AMX-Sb_w10_ (Figure 6b) obtained in under- and over-limiting current modes. The shape of the spectra for the pristine membrane and the sample contacted with wine are significantly different. These differences are observed in all three (high-, mid- and low-frequency) domains.

#### 3.3.1. The High-Frequency EIS Domain

**Figure 7:**
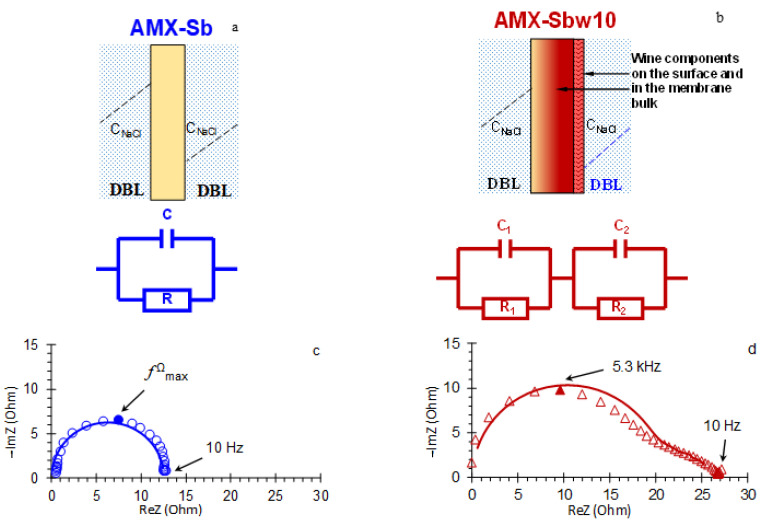
Electrical equivalent circuits for systems containing pristine AMX-Sb (**a**), AMX-Sb_w10_ (**b**) and adjacent DBLs. A high-frequency (10 Hz–1.3 × 10^5^ Hz) arcs of AMX-Sb (**c**) and AMX-Sb_w10_ (**d**) impedance spectra, obtained at $i/i_{\lim}^{theor}$= 0: experimental values are indicated by dots, approximations using EEC are indicated by solid lines. Explanations are given in the text.

Consider first the high-frequency (10 Hz–1.3 × 10^5^ Hz) domain. In the case of the AMX-Sb membrane, the spectra in this domain take the form of a semicircle (Figure 6a and Figure 7c). They can be approximated using the *RC* element (Figure 7a), where the resistance, *R*, is equivalent to the ohmic resistance of the membrane, its interphase boundaries and adjacent DBLs [47]; the effective electrical capacitance, *C*, is mainly controlled by the capacity of EDL on the interphase boundaries [47,54].

The difference between this equivalent circuit and those described in a number of works, for example, in [32,46,55,56], lies in the fact that it does not include the resistance of a solution located between the outer boundaries of the DBLs (where the concentration of the solution is equal to the concentration of the feed solution) and the measuring electrodes. This component is excluded from spectra at the stage of the experimental data-processing.

The impedance spectra of the AMX-Sb_w10_ in a similar frequency domain has a specific shape (Figure 6b and Figure 7d). Such a form of spectra, as a rule, is recorded in the presence of two layers, which are characterized by significantly different transport time constants [47,57,58]. A schematic representation of such a membrane system and its equivalent circuit is shown in Figure 7b. This circuit consists of two series-connected *RC* elements.

The first one, *R*_1_*C*_1_, describes the ohmic resistance and effective electrical capacitance of the membrane, its interphase boundaries and DBLs, which have changed as a result of fouling by the components of wine. 

**Figure 8:**
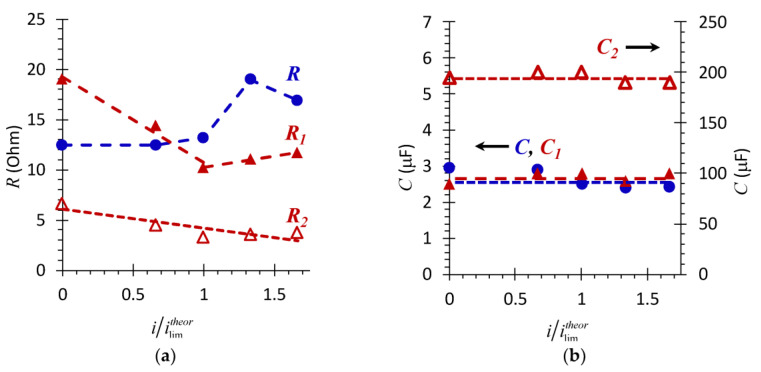
Dependence of the resistances (**a**) and effective electrical capacitances (**b**) of the AMX-Sb (*R*, *C*) and AMX-Sb_w10_ (*R*_1_, *C*_1_ and *R*_2_, *C*_2_) samples upon the direct current density normalized to the limiting current, calculated using Equation (1).

The second element, *R_2_C_2_*, characterizes the layer of wine components on the membrane surface. The example of experimental data approximation using these equivalent circuits is shown in Figure 7c,d. Figure 8 generalizes the results of such approximations for all the current densities applied.

The dependence of the resistance of the system with the pristine membrane (Figure 8a) upon the direct current density has a complicated form. In the underlimiting current modes, (0 < *i/i_lim_^theor^* < 1), *R* slowly increases with increasing current (Figure 8a). At currents close to the limiting value, the value of *R* increases sharply. This dependence in the underlimiting and close to the limiting currents is mainly determined by changes in the resistance of the DBLs, primarily the depleted one [47]. Indeed, in the range 0 < *i/i_lim_^theor^* < 1, there are no reasons for a change in the resistance of the system with AMX-Sb with increasing current. Therefore, the registered dependence of *R* on *i/i_lim_^theor^* is due to the concentration profiles formation (schematically indicated by the dashed line in Figure 7a,b) under conditions where the increase in the resistance of the depleted DBL is close to the decrease in the resistance of the enriched DBL.

At current densities close to *i/i_lim_^theor^*, the resistance of the depleted DBL greatly increases because of the emerging deficiency of charge carriers near the surface of the membrane. In the overlimiting current modes, water splitting begins at the membrane/solution boundary. This is evidenced, in particular, by the appearance of the pronounced Gerischer impedance (Figure 6a) on the impedance spectra of the pristine membrane. Protons, which enter the adjacent to the AEM solution, as well as the development of electroconvection, reduce the resistance of the depleted DBL [59]. The decrease in the values of *R* can also be promoted by competitive transfer through the membrane of hydroxyl ions: according to [60], the electrical resistance of the AEM (which is close to the membranes under study characteristics) decreases by a factor of 2 with the replacement of Cl^–^ counter ions by OH^–^ ions.

The calculated value of the effective capacitance, *C* = 0.62 μF cm^−2^ (2.5 μF), (Figure 8b), for the AMX-Sb membrane is consistent with the results obtained in work [56] for the AMX membrane. Our estimates show that the value of this capacitance remains practically unchanged with increasing current density (Figure 8b). Taking into account the fact that there is no contribution of the geometric capacitance of the DBLs in the investigated frequency domain [47], one can conclude that the charge of the AMX-Sb surface remains constant during the experiment. This means that the conditions where the EIS is obtained do not cause changes in the concentration or composition of the fixed groups at the membrane/solution interface, which are sometimes observed in the over-limiting current modes [61].

Measured in the absence of a direct electric current (*i* = 0), the total (*R*_1_ + *R*_2_) resistance of the AMX-Sb_w10_ after contact with wine is two times higher (Figure 7d) than that of the pristine membrane (Figure 7c). The contribution of the resistance of wine components layer on the AMX-Sb_w10_ surface, *R_2_*, to the total resistance is no more than 15%. However, its specific electric conductivity is two orders of magnitude lower than that of the membrane bulk, given that the thickness of this layer is only 2–3 μm (Figure 4b). The dependences *R*_1_, *R*_2_ on the direct current density (Figure 8a) differs markedly from those observed for the pristine AMX-Sb membrane. In the range of 0 < *i/i_lim_^theor^* ≤ 1, the value of *R_1_* for AMX-Sb_w10_ sample decreases rapidly. At currents close to the limiting value and higher, *i/i_lim_^theor^* ≥ 1, the values of *R*_1_ become lower than the values of *R* obtained for the pristine membrane. In the overlimiting modes, *R*_1_ gradually increases, and *R_2_* gradually decreases with increasing current density.

A decrease in *R*_2_, most likely, is due to a decrease in the thickness of the layer of wine components on the surface of the AMX-Sb_w10_ with an increase in time of the membrane stay under current. Apparently, in the superimposed electric field, anthocyanins, which contain positively charged chromophore groups (flavylium cations) and are retained in the layer formed only due to hydrogen bonds and van der Waals forces, move to a negatively charged cathode. The determining factor in reducing the thickness of the layer is not so much the current strength as the duration of the membrane stay under electric field.

The reduction in *R*_1_ in the under-limiting current modes and its smoother growth in the over-limiting modes in comparison to *R*, most likely, are due to the partial destruction of the complex colloidal structures that are formed by wine components in the pores of the AMX-Sb_w10_. The cause of their destruction, apparently, is the salting-out effect [62]. A similar effect is observed, for example, in protein solutions after the addition of certain electrolytes [63]. Such electrolyte in our case is NaCl. Note that some of the wine components (polyphenols, saccharides) appear in the 0.02 M NaCl solution at the stage of AMX-Sb_w10_ and AMX-Sb_w72_ preparation for electrochemical studies. Apparently, the application of an electric field contributes to the removal of the products of colloidal structures destruction. In addition, the high electric field strength suppressed the attachment of microorganisms to the AEMs surface [64]. As a result, the mobility of counterions in the membrane increases, and its resistance decreases.

As for the effective electric capacitances *C*_1_ and *C*_2_ (Figure 8b, Equation (2)), their values do not depend on the current density, just as in the case of the pristine membrane. Moreover, the value of *C_1_* for the AMX-Sb_w10_ is only slightly higher, while *C_2_* is two orders of magnitude higher than *C* calculated for the pristine membrane. The observed growth in *C_2_* seems to be associated with a significant increase in the roughness factor and in the real area of the AMX-Sb_w10_/solution interface due to appearance of spatial colloidal structures with distributed positive and negative charges [12]. It should be noted that some of wine components remain on the surface of AMX-Sb_w10_ after a sufficiently long time (more than 20 h) of the membrane operation under current. This is evidenced by the non-zero values of *R_2_* (Figure 8a) and high values of *C_2_* for *i/i_lim_^theor^* = 1.6 (Figure 8b).

#### 3.3.2. The Middle-Frequency EIS Domain

**Figure 9:**
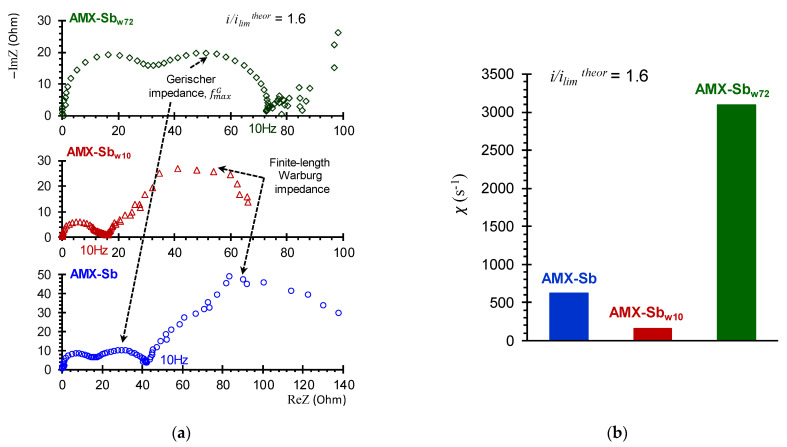
Electrochemical impedance spectra of the AMX-Sb, AMX-Sb_w10_, AMX-Sb_w72_ (**a**) and the values of the effective water splitting constants (**b**) found from the frequencies of the maximum points on the Gerischer arc (Equation (3)).

Apparently, foulants partially screen the catalytically active fixed groups on the surface of the membrane. As a result, the surface of AMX-Sb_w10_ loses the ability of water-splitting. This is evidenced by the absence of the Gerischer impedance arc on the impedance spectra of the AMX-Sb_w10_ (Figure 6b and Figure 9a), whereas in case of AMX-Sb (Figure 6a and Figure 9b), the arc appears at *i/i_lim_^theor^* > 1 and develops with an increase in current in the frequency range from 10 to 10^3^ Hz. For the AMX-Sb, the values of the effective water-splitting reaction constants, *χ* (Figure 9b), found from the frequency corresponding to the maximal value of imaginary component on the Gerischer impedance spectrum (Figure 9a), are in the range from 250 s^−1^ (*i/i_lim_^theor^* = 1.0) to 700 s^−1^ (*i/i_lim_^theor^* = 1.6). These values have good correlation with those found for similar membranes [35]. At the same time, the values of *χ* for the AMX-Sb_w10_ sample tend to zero (Figure 9b).

A longer (72 h) contact of the membrane with wine leads to an enhancement in water splitting: the Gerischer impedance arcs for AMX-Sb_w72_ sample increase noticeably as compared to AMX-Sb and AMX-Sb_w10_ (Figure 9a). The value of *χ* reaches 3100 s^−1^ (Figure 9b), eight times greater than those found for the AMX-Sb_w10_. The reason for the enhancement in water-splitting is, most probably, the appearance on the surface of AMX-Sb_w72_ of microorganisms (Section 3.2) that do not have time to proliferate on the surfaces of AMX-Sb and AMX-Sb_w10_ (Figure 4a,b).

**Figure 10:**
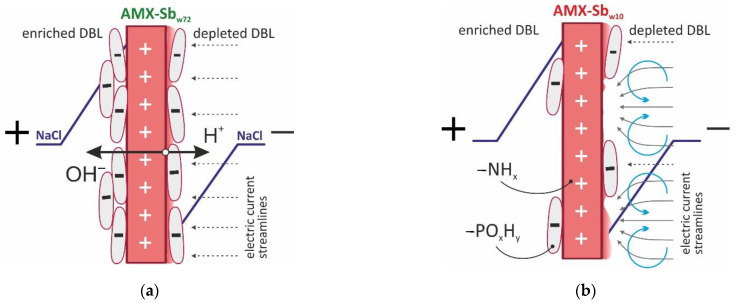
Schematic representation of the effect of foulant nature and its distribution over the membrane surface upon water-splitting (**a**) and electrovonvection (**b**).

It is known [65] that the bacterial outer membrane mainly consists of phosphatidylethanolamine, phosphatidylglycerol and diphosphatidylglycerol. Moreover, the deprotonation of phosphorus groups in the region of neutral pH values gives bacteria and other microorganisms a negative charge, which ensures their preferential adsorption on the surface of AEMs with positively charged fixed amino groups [66]. Therefore, biofouling leads to the formation of bipolar boundaries that facilitate the generation of H^+^ and OH^-^ ions under the action of an electric current (Figure 10a). T. Belloň et al. [67,68] observed a similar phenomenon after ssDNA sorption by the membrane surface.

The EIS was carried out in conditions where the membrane surface contacted with the wine was facing the desalination compartment of the electrodialysis cell. A high electric field strength, which is necessary for water splitting [69], could arise exactly at the bipolar boundary of this surface. This means that more intense biofouling on the receiving surface of AMX-Sb_w72_ could hinder the transport of salt ions through this membrane, i.e., promote an increase in the ohmic resistance of a fouled sample (Section 3.1), but did not have any effect on water-splitting.

#### 3.3.3. The Low-Frequency EIS Domain

Analysis of impedance spectra in the low-frequency (10 Hz–3 × 10^−3^ Hz) domain was carried out in order to assess the effect of changes in membrane surface properties after contact with wine on DBL thickness. In the case of AMX-Sb_w72_, the impedance spectra in this frequency domain have a significant dispersion (Figure 9a), which is caused by the effect of intensive water splitting. This is why the dependence of the depleted DBL thickness on the *i/i_lim_^theor^*, found from difference in values of the real component of Warburg impedance at high and low frequencies [37], was obtained for AMX-Sb and AMX-Sb_w10_ only.

**Figure 11:**
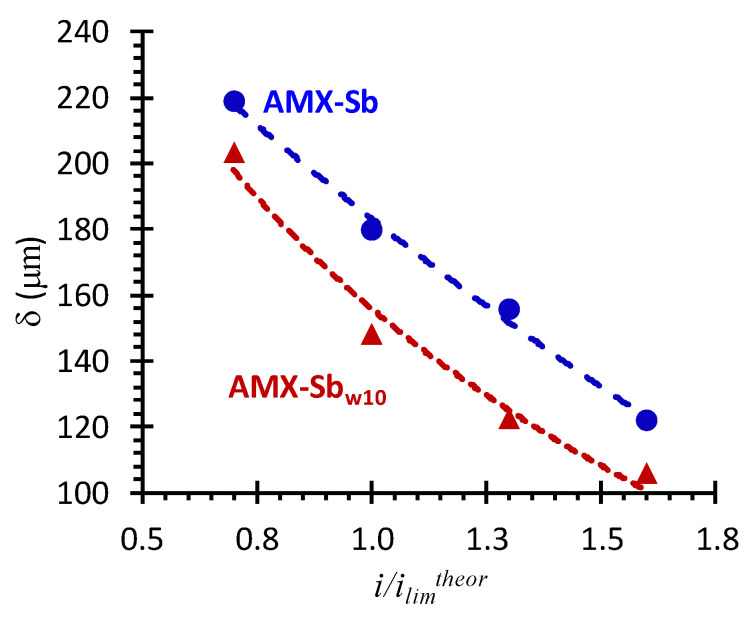
Dependence of the DBL thickness (δ) upon current density in systems with AMX-Sb (circles) and AMX-Sb_w10_ (triangles) membranes.

Figure 11 shows that in both cases (the pristine membrane and the membrane after contact with wine), there is a decrease in the DBL thickness with increasing current density. This decrease is observed at currents close to the limiting one and higher, and is more significant for the AMX-Sb_w10_. Our previous studies [59] allow one to conclude that under the conditions of the experiment (see Section 2), the reason for decrease in the depleted DBL thickness is electroconvection. Note that in the case of the AMX-Sb_w10_, the intensification of electroconvection takes place against the background of a decrease in the surface charge and its hydrophilization compared to the AMX-Sb (contact angels for AMX-Sb, AMX-Sb_w10_ and AMX-Sb_w72_ are equal to 58 ± 2, 50 ± 2 and 45 ± 2, respectively [12]). These conditions are directly opposite to those that contribute to the development of electroconvection [44,59]. Apparently, the determining factor for the enhancement of electroconvection in case of the AMX-Sb_w10_ is the growth in the inhomogeneity of the electric field, caused by the isle-type localization of the more hydrophilic but less conductive components of wine (Figure 10a) on a sufficiently hydrophobic pristine surface as well as the reduction in water splitting.

## 4. Conclusions

The analysis of the electrochemical impedance spectra obtained in the frequency range from 3 × 10^−3^ to 1.3 × 10^5^ Hz at zero-current density, as well as in under- and over-limiting current modes, is extremely informative for understanding the mechanisms of membrane fouling and identifying their consequences for the behavior of membranes in the applied electric field.

Analysis of the AMX-Sb_w10_ impedance spectra shows that the contribution of the wine components layer (on the membrane surface) resistance to the total resistance does not exceed 15%. However, its specific electric conductivity is two orders of magnitude lower than that of the membrane bulk, given that the thickness of this layer is only 2–3 μm.

With the increase in the duration of the AMX-Sb_w10_ operation in electrodialysis desalination of 0.02 M NaCl solution, the resistance of the membrane volume and the layer of wine components on its surface decreases. This is due to the partial destruction of the complex colloidal structures that are formed by wine components. The cause of their destruction, apparently, is the salting-out effect.

The value of electric capacitance of the foulant layer on the surface of AMX-Sb_w10_ is two orders of magnitude higher than that calculated for the pristine membrane. The observed growth seems to be associated with a significant increase in the roughness factor and in the real area of the AMX-Sb_w10_/solution interface due to the appearance of a spatial colloidal structures with distributed positive and negative charges.

These changes in the structure and chemical composition of the AMX-Sb_w10_ membrane surface lead to a reduction in water-splitting and an enhancement of electroconvection in the over-limiting current modes in comparison to the pristine membrane. Despite the noticeable hydrophilization of the AMX-Sb_w10_ surface as compared to AMX-Sb, electroconvection develops. This causes the decrease in the thickness of the depleted diffusion layer. The observed effect is apparently due to the isle-type distribution of anthocyanin-containing substances along the undulating surface of the investigated membrane.

The subsequent contact of the membrane with wine (72 h) leads to the formation of a fairly uniform layer, the mixture of wine components and microorganisms, on the AMX-Sb_w72_ membrane surfaces. The biofouling of AMX-Sb_w72_, led to an increase in water-splitting and the reduction in electroconvection in over-limiting current modes. The fact that biofouling and its negative effect on the behavior of the membrane system begins to manifest itself no earlier than 10 h later, allows for concluding that the cleaning-in-place procedure used in industry will be most effective when applied on a daily basis.

We hope that the demonstrated application of impedance spectroscopy will stimulate other researchers to use this method more widely, not only in the study of fouling, but also as a support for the design of membrane cleaning protocols.

## Figures and Tables

**Table 1:** Main physicochemical characteristics of the AMX-Sb membrane (experimental data).

Type	Homogeneous, Strong Base ^a^
Thickness in 0.02 M NaCl solution, μm	160 ± 10 ^b^
Conductivity in 0.02 M NaCl solution, S m^−1^	0.28 ± 0.02 ^b^
Ion-exchange capacity, meq g^−1^ (swollen membrane)	1.30 ± 0.05 ^b,c^
Water content, g H_2_O• (g dry membrane)^−1^	0.20 ± 0.05 ^b^
Membrane density, g cm^−3^	1.10 ^c^

^a^ Manufacturer data [38]. ^b^ Our measurements. ^c^ [39].
